# Control of Transcription Initiation by Biased Thermal Fluctuations on Repetitive Genomic Sequences

**DOI:** 10.3390/biom10091299

**Published:** 2020-09-09

**Authors:** Masahiko Imashimizu, Yuji Tokunaga, Ariel Afek, Hiroki Takahashi, Nobuo Shimamoto, David B. Lukatsky

**Affiliations:** 1 Cellular and Molecular Biotechnology Research Institute, National Institute of Advanced Industrial Science and Technology, Tokyo 135-0064, Japan; tokunaga.y@aist.go.jp; 2 Center for Genomic and Computational Biology, Department of Biostatistics and Bioinformatics, Duke University, Durham, NC 27708, USA; ariel.afek@duke.edu; 3 Medical Mycology Research Center, Chiba University, Chiba 260-8673, Japan; hiroki.takahashi@chiba-u.jp; 4 Molecular Chirality Research Center, Chiba University, Chiba 263-8522, Japan; 5 Plant Molecular Science Center, Chiba University, Chiba 260-8675, Japan; 6 National Institute of Genetics, Mishima, Shizuoka 411-8540, Japan; nshima@nig.ac.jp; 7 Department of Chemistry, Ben-Gurion University of the Negev, Beer-Sheva 8410501, Israel

**Keywords:** promoter sequences, repetitive sequences, pausing, abortive initiation, RNA polymerase, nonlocal base pair stability, thermal fluctuations

## Abstract

In the process of transcription initiation by RNA polymerase, promoter DNA sequences affect multiple reaction pathways determining the productivity of transcription. However, the question of how the molecular mechanism of transcription initiation depends on the sequence properties of promoter DNA remains poorly understood. Here, combining the statistical mechanical approach with high-throughput sequencing results, we characterize abortive transcription and pausing during transcription initiation by *Escherichia coli* RNA polymerase at a genome-wide level. Our results suggest that initially transcribed sequences, when enriched with thymine bases, contain the signal for inducing abortive transcription, whereas certain repetitive sequence elements embedded in promoter regions constitute the signal for inducing pausing. Both signals decrease the productivity of transcription initiation. Based on solution NMR and in vitro transcription measurements, we suggest that repetitive sequence elements within the promoter DNA modulate the nonlocal base pair stability of its double-stranded form. This stability profoundly influences the reaction coordinates of the productive initiation via pausing.

## 1. Introduction

In bacteria, transcription at a promoter is initiated by σ factor that forms a holoenzyme by binding to RNA polymerase (RNAP) core enzyme. The principal σ factor in *Escherichia coli* is termed σ^70^. *E. coli* promoters targeted for transcription initiation by σ^70^ holoenzyme have been characterized by having two consensus motifs approximately 10 and 35 bases upstream of the transcription start site (TSS). These motifs consist of a TATAAT (−10 box) and a TTGACA (−35 box), conserved in the promoters with high binding affinity to σ^70^ holoenzyme [1,2,3]. However, biologically functional promoters with high transcriptional activities usually do not have the full consensus motifs, but rather have nonlocal sequence signatures across the overall promoter region [4]. The reason for this is partially due to the need for RNAP to escape the promoter [5] but it has not been fully elucidated with respect to the regulatory mechanism of transcription initiation.

**Figure 1:**
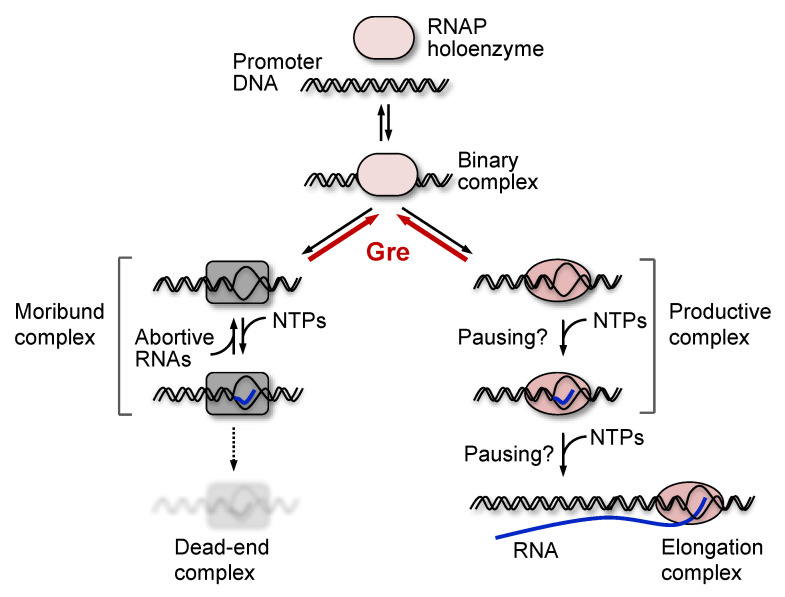
Branched initiation pathway. The action of Gre proteins at branches is shown by red color. In the presence of Gre proteins, the branching becomes reversible (thick red arrows) so that the moribund and the productive complexes can be exchanged each other [15]. Abortive RNA synthesis by the moribund complex is a slow process compared to full-length RNA synthesis (usually, up to 20 min [9,23]), which is reduced by Gre proteins, and thus is genome-widely detectable by RNET-seq with Gre-dependency of the data [25]. At several promoters, the moribund complex is further converted into a dead-end complex that still retains abortive RNA but has no elongation activity [8]. RNAP also pauses during productive initiation [10,11,12,23], which often involves backtracking of RNAP by one bp and thus can be resolved by Gre. Long-lifetime pausing by the moribund complex is incorporated in the processes of abortive transcription.

In transcription initiation, σ^70^ holoenzyme often synthesizes non-productive (abortive) short RNA [6,7]. When it occurs in a promoter, this process inhibits the productive initiation and the following elongation processes [8,9]. During abortive synthesis, a ternary initiation complex of the σ^70^ holoenzyme starts transcription and then backtracks to shorten the RNA–DNA hybrid, which releases short RNAs [10,11,12,13]. Such a complex is termed moribund complex (see review [14]). The initiation pathway leading to the abortive synthesis by the moribund complex is branched from the pathway leading to full-length RNA synthesis by a productive complex [8]. This branch can be a mechanism controlling transcription initiation by RNAPs of *E. coli* and other bacteria [15,16,17] (Figure 1). The binary moribund complex can be converted into the productive complex by binding of allosteric effectors like Gre proteins to the complex [18]. This function of the Gre proteins is different from their well-known function, i.e., cleavage of the 3′ RNA that is extruded from the active center of the backtracked polymerase [19]. The level of abortive initiation depends on sequences both upstream and downstream of the TSS [20,21,22]. The first ∼20 bp of the downstream sequence is termed initially transcribed sequence (ITS).

When it occurs, pausing on the pathway to productive initiation delays transcription kinetics on a physiological timescale by affecting promoter escape [10,11,12,23]. It has been reported that specific sequences in ITS can induce pausing during initiation [21,24]. Therefore, not only the moribund complex, but also RNAP pausing of the productive complex can provide sequence-specific mechanisms controlling transcription during initiation. To date, individual sequence signals that are responsible for either abortive synthesis or pausing have not been separately identified.

Previously, we identified a highly conserved sequence motif that induces elongation pausing in *E. coli* [25]. This motif impedes forward translocation of RNAP, as well as the following NTP addition [25]. However, our later analysis revealed that the presence of the conserved sequence motif alone is not solely responsible for RNAP pausing [26]. In particular, we demonstrated that, during elongation pausing, repetitive sequence elements can increase the magnitude of diffusive backtracking of RNAP on the DNA upstream of the pausing site, thereby generating a large variation in the lifetimes of RNAP pausing at the conserved pausing motifs [26]. The driving force responsible for this effect stems from thermal fluctuations of molecules, i.e., entropy or diffusion, shaped by the presence of repetitive DNA sequence elements [26]. Thermal fluctuations can significantly affect the mechanism of interaction between RNAP and DNA, allowing multiple macromolecular conformations (termed conformational heterogeneity) to contribute to the resulting functional output, such as, for example, RNAP pausing. This finding allowed global prediction of elongation pausing in *E. coli*.

In this study, using a similar approach, we characterized abortive transcription and pausing during initiation in *E. coli* at a genome-wide level. Our results suggest that a T-rich signal located in ITS can induce abortive synthesis, while repetitive sequences distributed in promoter regions can be a signal inducing pausing. We propose that the nonlocal base pair stability of double-stranded DNA (dsDNA) is the possible physicochemical feature that affects the reaction coordinates of a productive initiation via pausing.

## 2. Materials and Methods

### 2.1. Mapping of Short Read Sequences to TSS

**Figure 2:**
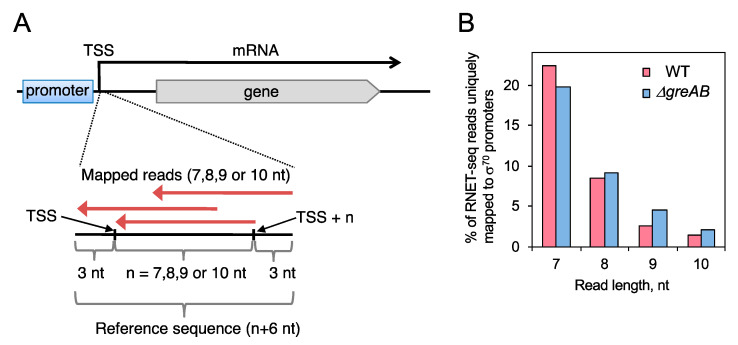
RNET-seq analysis for abortive transcription and pausing during initiation. (**A**) The short RNET-seq reads of a fixed length (7, 8, 9 or 10 nt long) were mapped to the TSS downstream of 775 σ^70^ promoters [27]. In the mapping procedure, we allowed ±3 nt positional fluctuations of TSS. Since the 7–10 nt reads were too short to be uniquely and precisely mapped to the entire *E. coli* genome, we extracted the TSS downstream sequences from the genome as reference sequences enabling us to uniquely map these short reads to the reference sequence library (see Materials and Methods for more details). Mapped short reads with the direction opposite to transcription are schematically shown by arrows. Using this procedure, (**B**) The short reads of each length, with sense orientation to mRNA genes, were mapped to the special reference by Blat program [28]. The uniquely mapped reads with perfect matches were selected for the analysis performed in the present study. The reads were obtained by RNET-seq of the nascent RNAs of *E. coli* WT and Δ*greAB* cells [25].

In the RNET-seq method, RNAP basically protects 14-nt or longer RNA from RNase digestions during elongation [25]. Thus, many of these <14-nt reads are predicted to be derived from the RNAs associated with the ternary complexes in the stage of initiation or the transition to elongation (i.e., such <14-nt reads should be mainly mapped to just downstream of TSS). However, the RNET-seq reads shorter than 14 nt are not uniquely mapped to the *E. coli* genome (i.e., mapped to multiple genomic locations) [25]. In order to prevent being mapped to multiple genomic locations other than just downstream of TSS, the reference genomic sequence was confined only to the vicinity of TSS. In particular, we compiled the reference sequence library that only contains sequences within genomic positions confined from 3nt upstream to *n* + 3nt downstream from the TSS, where *n* is the read length in a range of 7, 8, 9, or 10 nt (Figure 2A).

The TSS of 775 σ^70^ promoters were used for the reference, which are provided by RegulonDB, with the high reliability based on the experimental identification and validation [27]. The short reads of each length with sense orientation to mRNA genes were mapped to the special reference. The mapping was performed using Blat v35 with the parameters -minScore = 0 -tileSize = 6 -stepSize = 1 [28]. Among those uniquely mapped reads, only 20–35% of them started exactly at TSS (Supplementary Table S1). This suggests that the ±3 nt positional fluctuations of TSS that we assumed correctly grasp the distribution of TSS in vivo, or otherwise are likely due to trimming of adapter sequences from these short reads during sequence processing [25]. We also verified that the reference created by us can efficiently prevent nonunique mappings even within the TSS vicinity: the number of the uniquely and nonuniquely mapped reads were almost the same in the 9-nt and 10-nt reads and less than two-fold difference in the 7-nt and 8-nt reads (Supplementary Table S1).

### 2.2. Calculation of FEINC

The free energy index for the non-consensus mode of protein–DNA binding (FEINC) was computed as described previously in Reference [26]. Briefly, to calculate FEINC we generate an ensemble of random DNA binders. We assume that a model protein (random binder) makes ***M*** base-pair contacts with the DNA, and we used ***M*** = 8. We also assume that the protein–DNA interaction energy at each genomic position ***i*** is a sum of ***M*** interaction energies:

(1) $$U\left(i\right)=\overset{M+i-1}{\underset{j=i}{\sum }}\overset{\;}{\underset{\alpha =\left(A,T,C,G\right)}{\sum }}K_{\alpha }s_{\alpha }\left(j\right),$$

where ***s**_**α**_*(***j***) represents the elements of a four-component vector of the type (**δ***_**α**_*_***A***_, **δ***_**α**_*_***T***_, **δ***_**α**_*_***C***_, **δ***_**α**_*_***G***_) and **δ***_**α**__**β**_* = 1 if ***α*** = ***β*** or **δ***_**α**__**β**_* = 0 if ***α*** ≠ ***β***. For example, if the ***A*** nucleotide is positioned at the genomic coordinate ***j***, then this vector takes the form (1,0,0,0). In order to generate each model protein (random binder), we draw the values ***K_A_***, ***K_T_***, ***K_C_***, and ***K_G_*** from Gaussian probability distributions, ***P***(***K***_***a***_), with zero mean, $<K_{\alpha }>=0$, and standard deviation $\sigma_{\alpha }=2k_{B}T$, where *k*_B_ is the Boltzmann constant, and ***T*** is the temperature. The chosen energy scale, $2k_{B}T\approx$ 1.2 kcal/mol, represents a typical strength of a hydrogen bond, or an electrostatic bond that a protein makes with one DNA base pair. We have shown previously that the resulting free energy is robust with respect to the choice of model parameters [26,29]. For each random binder, we define the partition function within the sliding window of width ***L*** base pairs:

(2) $$Z=\overset{L+1-M}{\underset{i=1}{\sum }}\exp\left(-U\left(i\right)/k_{B}T\right),$$

and we used ***L*** = 30 in our calculation. The resulting free energy of protein–DNA binding in this sliding window:

(3) $$F=-k_{B}T\;\ln\left(Z\right).$$

We then assign the computed ***F*** to the sequence coordinate in the middle of the sliding window. We repeat this procedure for an ensemble of 250 model random binders (where each random binder is uniquely characterized by four random numbers: ***K*_*A*_**, ***K*_*T*_**, ***K*_*C*_**, and ***K*_*G*_**) and compute the average free energy, <F>, with respect to this ensemble. Finally, we normalized <F> to get the free energy per base-pair contact f=<F>/M. We term this normalized free energy, ***f***, the free energy index for the non-consensus mode of protein–DNA binding (FEINC).

### 2.3. Calculation of the P-Value

In order to compute the *p*-values, evaluating the statistical significance of the difference between the high ratio (X ≥ 2) and the low ratio (X ≤ 0.5) subgroups (see below for details), we used the following procedure. First, we unite all the sequences belonging to the two original subgroups into one group, and randomize the order of the sequences. As a result of this procedure, we obtain one randomly mixed group of sequences. Next, by randomly selecting sequences from the mixed group, we compile two new randomized subgroups of sequences, each subgroup containing exactly the same number of sequences as in the original subgroups. We then compute the average value of FEINC within the interval (−30,30)-nt for each of the randomized subgroups. If the absolute value of the difference between these two values is equal or larger than the corresponding original difference, we count this randomized trial as a successful trial. We repeat the entire procedure 10^5^ times. Finally, we define the *p*-value as the ratio of the number of the resulting successful trials to the total number of trials.

### 2.4. In Vitro Transcription

*E. coli* RNAP holoenzyme, GreA and GreB proteins, which were purified as described in Refs [30], were kindly gifted by Dr. Kashlev’s laboratory. NTPs and oligonucleotides were purchased from GE Healthcare and Fasmac, respectively. The liner DNA templates from −77 to +63 when TSS is +1, each of which contains one of 9 promoters (see Section 3.2), was prepared by PCR using oligonucleotides (see Table S2 for the full sequences). These DNA templates were purified by PAGE.

All reactions were performed in transcription buffer (TB; 20 mM Tris–HCl, pH 7.6, 5 mM MgCl_2_, 1 mM 2-mercaptoehanol, 0.1 M KCl) at room temperature. The holoenzyme (200 nM) and DNA template (10 nM each of 9 promoter DNA) were preincubated for 10 min in TB. Where present, GreA and GreB proteins were added to the holoenzyme at final concentration of 7 μM and 4 μM, respectively. Reaction was started by adding 100 μM NTPs at final concentration. Heparin (250 μg/mL) was added together with the substrates to eliminate enzyme turnover, which assures single-round reaction. After incubation for 1.5 min or 20 min, reaction was stopped by adding phenol/chloroform/isoamyl alcohol (25:24:1). The experiments with the presented results in this study were repeated twice and the represented ones are shown.

RNA transcripts were analyzed by Illumina sequencing. Briefly, cDNA libraries were constructed according to Ref [25]. The cDNAs were quantified by using a real-time PCR system CFX96 (Bio-Rad, Hercules, CA, USA). Illumina sequencing was performed with MiniSeq High Output Kit (75 Cycles). A typical output of the sequencing was ~3 × 10^6^ reads per sample. The sequencing reads were mapped and aligned to DNA template (Table S2) using Bowtie2 v.2.3.4.1 with default parameters [31]. The number of 3′ RNAs at every nucleotide position of the DNA template was counted as described previously [25].

### 2.5. Solution NMR

DNA oligonucleotides that were purified by reverse phase cartridge were purchased from Fasmac. Each double-stranded DNA (dsDNA) of 50 μM was generated in a 250 μL solution consisting of 90% H_2_O/10% D_2_O solvent with 20 mM Tris-D11 (pH 7.6 at 25 °C), 5 mM MgCl_2_, and 50 mM KCl, which was transferred into a 5-mm microtube (Shigemi, Tokyo). NMR experiments were performed on an Avance 700 spectrometer (Bruker, Billerica, MA, USA) equipped with a 5-mm TXI triple resonance probe at 15, 25, and 35 °C. Proton one-dimensional spectra were recorded with a 22 ppm spectral width, centered at 4.7 ppm, using the WATERGATE building block for solvent suppression. Sodium 3-(trimethylsilyl)-1-propanesulfonate was used as an external chemical shift standard. Free induction decays (FIDs) were acquired for 133 ms with 2048 digital points. The FIDs were accumulated 4096 times with interscan delays of 4.0 (15 °C) and 2.5 s (25 and 35 °C). Raw FIDs were multiplied by a cosine window function and Fourier transformed to frequency domain data, followed by phase correction and baseline correction. Signals in the chemical shift range of 11.6–14.2 ppm were integrated, which we used as the imino proton signal intensity.

## 3. Results

We have previously developed the RNase-footprinting followed by NET-seq (RNET-seq) method to identify the complexes that are paused during transcription elongation in *E. coli* wild type (WT) and in an isogenic strain deficient in genes for GreA and GreB (Δ*greAB*) [25]. Briefly, *E. coli* cells were rapidly lysed and any transcribing RNAPs were released from the genomic DNA and co-transcriptional translation by digestion with DNase I and RNase A, respectively. All RNAPs including those associated with the fragmented dsDNAs and their 5′-truncated nascent RNAs were immobilized on Ni^2+^-NTA beads via the histidine-tagged β’ subunit and then washed. The 5′ ends of the transcripts in the ternary complexes were trimmed with RNase T1/V1 to leave a minimal length of RNA protected by RNAP. The RNases were removed by further washing. Elution with imidazole released ternary complexes carrying ~6–30 nt long transcripts. Sequencing of the complementary DNA libraries produced ~2.67 × 10 ^7^ and ~2.60 × 10^7^ reads of 6–30 nt lengths for the WT and Δ*greAB* strains, respectively [25], which were used in this study.

In RNET-seq method, the longer the RNAP occupies a particular DNA site during elongation, the stronger the pause is detected at this DNA site. In addition, the different nascent RNA lengths correspond to the different states of the ternary complexes. Namely, during elongation, post-translocated, pre-translocated, and backtracked complexes protect 14-nt, 15-nt and >15-nt lengths of the RNA, respectively [25,32]. The detected RNAs shorter than 14 nt basically mean the nascent RNA that has not yet been extended to be 14 nt, i.e., such RNAs were associated with initially transcribing complexes. Therefore, this method may allow us to collect 6~13 nt long abortive RNA transcripts retained (prior to their release) in the moribund complex, in addition to RNA transcripts retained in a paused productive complex. In this scenario, the 5′ end of the unreleased abortive transcripts could be mapped at TSS and the corresponding number of reads was increased in the Δ*greAB* cells as compared with WT cells (Figure 1). We thus mapped and aligned short RNET-seq reads to TSS and the close vicinity of TSS in 775 σ^70^ promoter sequences by compiling a reference sequence library comprised of short sequences just downstream of TSS [27] (Figure 2A, also see Materials and Methods). The relative fraction of the uniquely mapped reads for 7~10 nt transcripts to these 775 promoters were similar between the WT and Δ*greAB* strains (Figure 2B). Since the two strains share almost the same number of the 6–30-nt (near total) reads, we directly compared the number of the uniquely mapped reads per promoter between the WT and Δ*greAB* strains in the following analysis, without normalization.

We then classified those σ^70^ promoters into the following three groups according to the magnitude of the ratio, *X*, of the amounts of nascent RNA transcripts (nrt) in Δ*greAB* cells, *nrt*(Δ*greAB)*, to that in WT cells, *nrt*(WT), respectively, *X* = *nrt*(Δ*greAB)* /*nrt*(WT). We always define this ratio, *X*, separately for each transcript length, 7nt, 8nt, 9nt, and 10nt, respectively. We term the three groups as increased ratio (*X* ≥ 2), similar ratio (0.5 < *X* < 2), and reduced ratio *(X* ≤ 0.5), respectively. Since Gre factors are unlikely to affect binding of RNAP holoenzyme to specific promoter sequences [19,33], we assume that only the first promoter group (*X* ≥ 2) possesses much abortive synthesis, while the second (0.5 < *X* < 2) and third (*X* ≤ 0.5) groups possess little abortive synthesis. Transcripts belonging to the third group may originate due to indirect influence or unknown functions of Gre factors. In other words, we suggest that abortive synthesis is predominately represented by the first group, but we do not suggest that all the transcripts in this group are abortive transcripts. As we mentioned above, pausing during productive initiation is also classified in the first group when the pausing involves backtracking. Thus, we hereafter term the first group abortive/pausing-enriched group.

Next, we investigated the group-specific sequence properties in terms of the following two different binding modes: (i) specific RNAP-DNA binding on consensus DNA motifs and (ii) nonspecific RNAP-DNA binding on repetitive DNA sequence elements. We assume here that these two types of binding mechanisms are entirely decoupled, i.e., the specific binding mechanism (i) does not affect the nonspecific binding mechanism (ii) and vice versa. Hereafter, we term the former mechanism as consensus mode of RNAP-DNA binding, and the latter mechanism as non-consensus mode of RNAP-DNA binding, respectively. The consensus mode conventionally assumes a single (or a few) dominant conformation(s) in the complex. This effect is often represented by information content, the level of sequence conservation within the motif defined [34]. The non-consensus mode assumes many conformations of the complex that can change as a result of thermal fluctuations. This effect can be modeled as one-dimensional diffusion of RNAP on DNA induced by repetitive DNA sequence elements [35]. In particular, in our recent works, we have developed a statistical mechanical modeling approach to take into account the effect of certain repetitive DNA sequence elements on protein–DNA binding free energy [36,37]. We have shown in these works that certain repetitive non-consensus genomic background sequences surrounding a consensus motif can significantly modulate binding of the target protein to DNA via the entropy dominated mechanism [36,37]. We have quantitatively characterized this mechanism using an equilibrium statistical mechanics model without fitting parameters, where actual genomic DNA sequences constitute the only input parameter [36,37]. This statistical concept has allowed us to quantitatively characterize microscopic heterogeneity of protein–DNA complexes stemming from thermal fluctuations as entropy-dominated free energy, which strongly depends on certain repetitive DNA sequence elements being recognized by a protein [36,37,38]. We term this free energy index for the non-consensus mode of protein–DNA binding (FEINC). We describe in detail the calculation of the FEINC in Materials and Methods. Using this approach, we have previously predicted that repetitive genomic sequences significantly enhance RNAP pausing during elongation by increasing the number of the paused complex conformations induced by thermal fluctuations [26]. Such a prediction was experimentally verified by observing enhanced diffusive backtracking of *E. coli* RNAP in genomic pause sites that are enriched with repetitive sequence elements [26].

### 3.1. The Significance of a Consensus Mode of RNAP-DNA Binding

**Figure 3:**
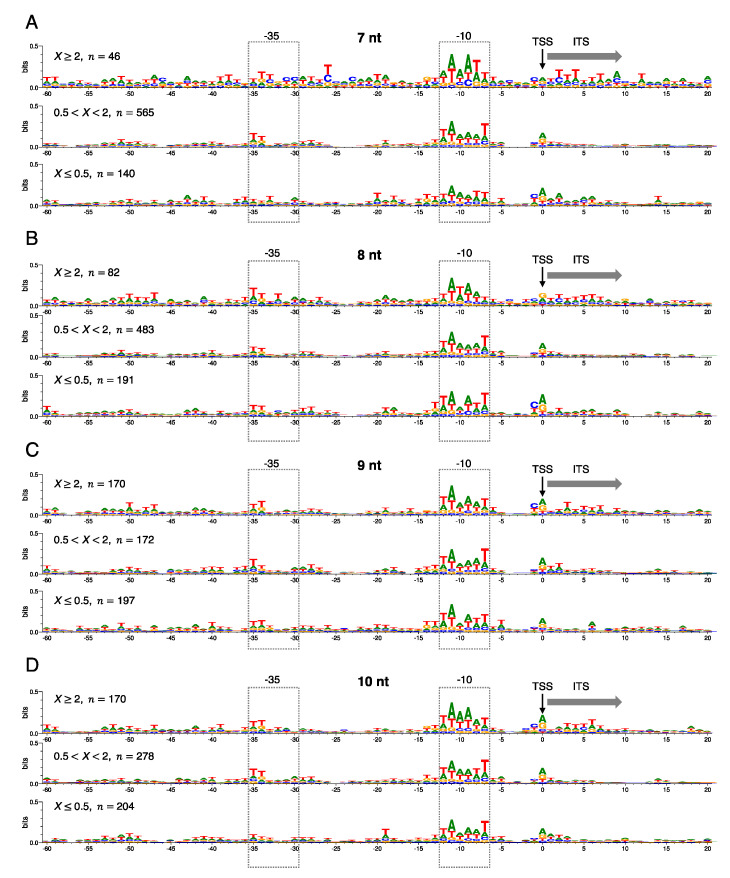
The effect of consensus mode of holoenzyme-DNA binding on initiation complexes having nascent RNAs of 7 nt (**A**), 8 nt (**B**), 9 nt (**C**), and 10 nt (**D**), respectively. The consensus effect is classified into the three promoter groups where abortive synthesis or pausing was decreased (*X* ≥ 2) (top), unaffected (0.5 < *X <* 2) (middle) and increased (*X ≤* 0.5) (bottom) by Gre proteins. Here *X* represents *nrt*(Δ*greAB*)*/nrt*(WT) ratio for the RNET-seq reads of each length that is mapped to the close vicinity of TSS of σ^70^ promoters; *n* represents the number of promoters composing the group. We have excluded rRNA promoters and promoters for unexpressed genes from these 775 σ^70^ promoters. The sequence conservation (information content, bits) in the promoter DNA region (from −60 to + 20, where TSS is +1) is represented by Sequence Logo [34]. −10 and −35 motifs are shown by boxes. TSS and ITS are shown by a vertical arrow and by a lateral arrow, respectively.

We found that only one of the 775 promoters (*metY* gene) has a complete match to the consensus motifs −10 (TATAAT)/−35 (TTGACA). When the promoter sequences were simply aligned based on TSS without gaps, the −10 and −35 motifs are not well conserved (information contents < 0.5 bits) through the complexes carrying the four different lengths of RNA (Figure 3). Notably, we found that approximately 6 consecutive T bases are slightly conserved in ITS in the abortive-pausing-enriched group (Figure 3, *X* ≥ 2). It has been reported that T bases in ITS stimulates abortive synthesis or pausing during initiation via biasing the translocation equilibrium of RNAP toward the pre-translocated state [20,21]. The bias to the pre-translocated state increases probability of backtracking of the RNAP relative to RNA-DNA hybrid, thereby being able to induce abortive transcription. Backtracking can be also induced by the unstable U-dA base pairs within the RNA-DNA hybrid that is encoded by the consecutive T bases [39,40,41]. Therefore, the T-rich ITS signal could be a candidate of a sequence signal to induce a process of abortive transcription in vivo, although it is not clear whether this signal is included in the consensus RNAP-DNA binding effect. More details about the relation between T-rich ITS and RNAP backtracking are discussed below.

**Figure 4:**
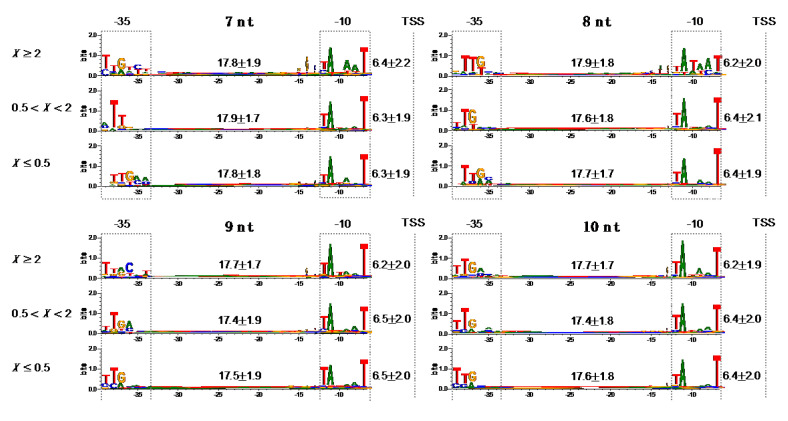
The consensus −10 and −35 motifs that are detected by considering variable spacer lengths. We used the same classification of the three promoter groups (*X* ≥ 2, 0.5 < *X* < 2 and *X* ≤ 0.5) and initiation complexes having 7–10 nt nascent RNAs, as the classification described in the legend of Figure 3. The bipartite motif search in σ^70^ promoter regions (−40 to −1, where +1 is TSS) were performed by Dipartite program [42] (see Figure 3 for the number of samples *n* in each group), in which the range of the spacer length between −10 and 35 motifs (both 6 bases) was defined as 15 to 21 bases. In order to find best motifs, the search process was repeated fifty times. The −10/−35 motifs and TSS are shown by boxes and lines, respectively. Sequence Logos represent the alignments with gaps to make uniform 21-base spacers between −10 and −35 motifs. The actual distributed lengths of the spacers between −10 and −35 motifs, and between −10 motif and TSS are shown as the average values ± standard deviations, together with gray arrows. The promoter sequences that were aligned to have the ≥20-nt long spacer between −10 motif and TSS were removed from the measured values of the spacer lengths, since such situations are structurally unlikely [45].

We next explored −10 and −35 motifs in the 775 promoters by regarding them as bipartite motifs that are separated by gap sequences with variable lengths [42]. We considered variable spacer lengths between the −10 and −35 motifs, and between −10 motif and TSS for the bipartite motif search, since these spacers affect both productive and abortive initiations [22,43,44]. This approach allowed us to find much better conserved −10 and −35 motifs (Figure 4, information contents ≳ 1.0 bits). Particularly, −11A and −7T bases of the −10 motif were the best conserved through the groups with the different *X* = *nrt*(Δ*greAB*)/*nrt*(WT) ratio and the different RNA lengths. This observation is consistent with the previous structural study showing that base-specific interactions between σ factor and promoter DNA occur primarily with −11A/−7T bases, which stabilize the RNAP-promoter-open complex [45]. The most prominent feature specifically observed in *X* ≥ 2 group is the relatively high integrity of the 6-bp motifs especially for the shorter 7-nt and 8-nt RNA complexes, in which almost complete consensus −35 and −10 motifs were detected in their complexes, respectively (Figure 4). This high integrity might be involved in increasing specific affinity of σ to the DNA motifs, which is also related to pausing or abortive initiation and their dependency to Gre proteins as discussed below. We could not detect significant differences in the spacer lengths among above-mentioned groups and complexes (Figure 4).

Taken together, the consensus −10/−35 motifs undoubtedly determine the strength of the holoenzyme-promoter-DNA binding as shown before [46], but are not enough to determine the fate of transcription initiation. We suggest that variations within the −10/−35 motifs as well as the consecutive T repeats within ITS may affect initiation pathways that depend on Gre proteins.

### 3.2. The Significance of a Nonconsensus Mode of RNAP-DNA (RNA-DNA Hybrid) Binding

**Figure 5:**
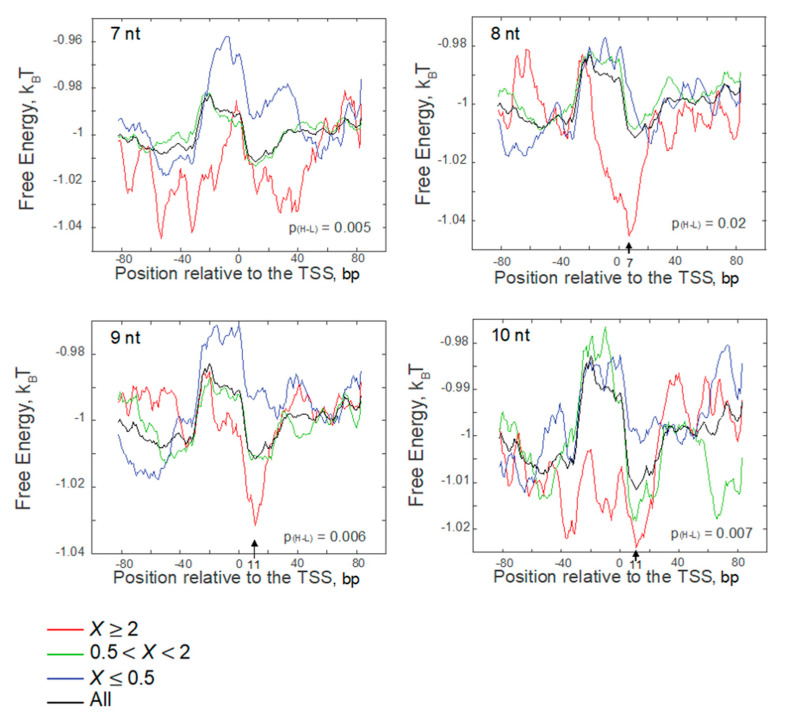
The free energy index for the nonconsensus mode of RNAP-DNA binding (FEINC) was reduced for the initiation complex in the vicinity of TSS. Here we grouped the sequences into three groups according to the ratio, *X* = *nrt*(Δ*greAB*)/*nrt*(WT), for the RNET-seq reads with the length of 7 nt, 8 nt, 9 nt, and 10 nt, respectively, mapped to TSS of each σ^70^ promoter. FEINC was calculated as described in Materials and Methods. In each plot for 8 nt, 9 nt, and 10 nt, a base position that has the lowest free energy is shown with an arrow. The computed *P*-values (H-L) evaluate the statistical significance of the difference between the high ratio (X ≥ 2) and the low ratio (X ≤ 0.5) groups. The calculation of these *P*-values is described in Materials and Methods.

The sequence effect represented by the average FEINC was significantly different between the abortive/pausing-enriched group (*X* ≥ 2) and the abortive/pausing-depleted group (*X* ≤ 0.5) (Figure 5). The former group (*X* ≥ 2) is characterized by the low FEINC for 7~10-nt transcripts. Interestingly, in the complexes retaining 8–10-nt nascent RNAs, the FEINC of the former group (*X* ≥ 2) is the lowest at the site with the position shifted towards the 3′ end of the nascent RNAs. This FEINC landscape appears to indicate an enhanced sliding of RNAP along DNA (i.e., backtracking relative to the RNA-DNA hybrid in the case of the ternary complex), according to our previous characterization of elongation pausing [26]. Likewise, the prevention of backtracking is predicted from the high average FEINC (i.e., high energy barrier to backtracking) upstream of TSS in the opposite group (*X* ≤ 0.5) for the complexes retaining 7-nt or 9-nt nascent RNA (Figure 5) [26].

**Figure 6:**
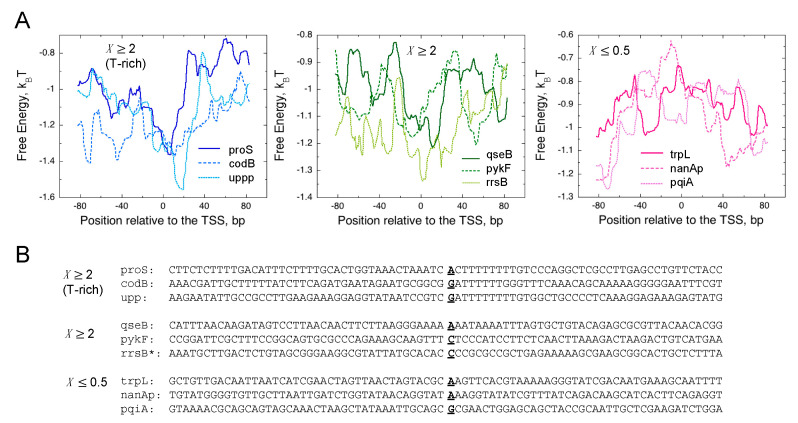
The relation between the FEINC (free energy) landscapes (**A**) and DNA sequences (**B**), representing examples of sequences from the three groups characterized by the ratio *X* = *nrt*(Δ*greAB*)/*nrt*(WT). The first group corresponds to *X* ≥ 2 with T-rich signal, the second group corresponds to *X* ≥ 2 without T-rich signal, and the third group corresponds to *X* ≤ 0.5, respectively. Each group has three representative promoters in which TSS of the panel B is indicated by bold font with underline. Note that the rrsB promoter, indicated by asterisk, has *X* = 3.81 but was excluded from sequence analyses shown in Figure 3, Figure 4 and Figure 5 due to the redundant property of the rRNA gene.

RNAP backtracking has been proposed for abortive RNA release from the secondary channel [14]. The model is so far consistent with the biochemical/biophysical results obtained by various research groups [10,11,12,14]. As mentioned above, backtracking is also predicted by the T-rich signal in ITS [20,21]. In fact, slight enrichment of the homopolymeric T in ITS is observed only in the abortive/pausing-enriched group (Figure 3, *X* ≥ 2). Examples of the FEINC for nine individual promoters are shown in Figure 6. We selected those examples from the three representative groups of DNA sequences. The first and second groups correspond to *X* ≥ 2, with and without the T-rich signal in ITS, respectively, and the third group corresponds to *X* ≤ 0.5 (Figure 6). The first and second groups exhibit a qualitatively similar FEINC landscape within −40 bp to +40 bp (a valley in the region (0, + 20) bp around TSS is observed). Such low FEINC stems from the presence of repetitive sequence elements in the *X* ≥ 2 group. Thus, repetitive sequences can accelerate reaction pathways via the backtracking of RNAP on DNA, including abortive synthesis and pausing. Higher FEINC within the region −40 bp to +40 bp in the *X* ≤ 0.5 group is obtained by less repetitive sequence elements.

**Figure 7:**
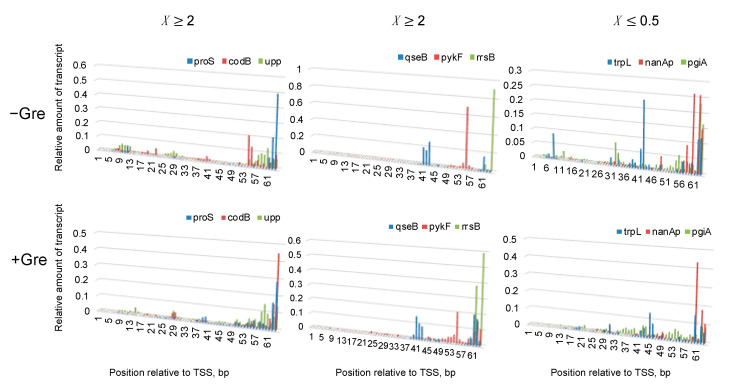
In vitro single-round transcription from 9 different promoters shown in Figure 6B. Entire transcription profiles at 20 min incubation with NTPs are shown in the presence (bottom) or absence (top) of GreAB. See the legend of Figure 6 for the categorization of promoters.

We then question (i) whether both the enrichments of >40 bp repetitive sequences and ~6-bp T-rich ITS encode the same signal allowing backtracking, or (ii) whether such respective sequence signals affect differently abortive synthesis and pausing. In order to address these questions, we performed single-round transcription assay using purified *E. coli* RNAP, GreAB proteins and linear 140-bp DNA templates for nine promoters. The results were analyzed by high-throughput sequencing of total RNA. The sequences of the nine promoters are shown in Figure 6B: three of six promoters of the abortive/pausing-enriched group (*X* ≥ 2) possess the T-rich ITS, while the other three do not possess the T-rich ITS. The remaining three promoters belong to the opposite group (*X* ≤ 0.5). By 20 min incubation with 100 µM NTPs, more 6–13-nt transcripts were produced by the presence of T-rich ITS in the former group, and the number of those short transcripts were reduced by GreAB, as expected for abortive initiation (Figure 7). Furthermore, the fraction of 6–13-nt transcripts increased time-dependently as predicted from the branched initiation pathway where slow abortive synthesis continues to occur long after completion of full-length RNA synthesis (Figure 1 and Supplementary Figure S1). Thus, our in vitro analysis showed that T-rich ITS induces abortive transcription. We discuss more details about these in vitro results in Supplemental Information.

**Figure 8:**
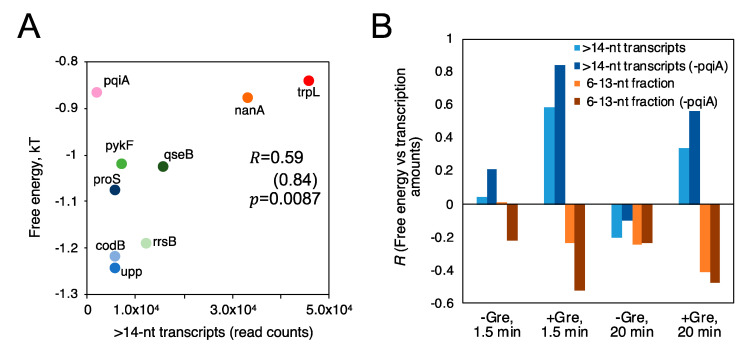
FEINC may predict the productivity of transcription initiation in vitro. (**A**) Positive correlation of the average FEINC (shown as free energy), which was computed for individual promoters within the interval (−40 bp to +40 bp) around TSS, with the number (read counts) of long >14 nt transcripts. Color code of 9 promoters is same as that of Figure 6A. Pearson correlation coefficient *R* between the two variables is shown. *R* values except pqiA are also shown in parentheses as well as the *p*-value. (**B**) GreAB and incubation time with NTPs alter the positive and negative correlation trends of the free energy (FEINC) with the number of >14-nt transcripts and the relative fraction of short 6–13 nt transcripts, respectively.

We further explored a relation between FEINC and transcription initiation from the nine different promoters in vitro. We found that the FEINC of these promoters tends to positively correlate with the number (i.e., the read count) of long >14-nt transcripts, when the pqiA promoter is excluded as an outlier (Figure 8A). The opposite (i.e., negative correlation) trend was observed in the relative fraction of short, 6–13-nt transcripts (Figure 8B). In order to obtain such correlations, we added Gre proteins and shortened the reaction time (1.5 min) (Figure 8B). Under these conditions, the abortive synthesis by moribund complex (typically >1.5 min) should be decreased or negligible, since Gre proteins enable switching of the complex into the productive complex by lowering the high activation energy to the pathway branched in these two complexes (Figure 1 and Supplementary Figure S1) [14,15]. Thus, we interpreted the low FEINC as indicating pausing of the productive complex rather than abortive synthesis of the moribund complex. The exceptional behavior of pqiA promoter appears to be consistent with its belonging to *X* ≤ 0.5 group, especially for the high FEINC over −40 bp to +40 bp. It is likely that the high FEINC may contribute to generate the exceptionally high activation energy to the branched pathway, making it less dependent on Gre proteins.

The pausing of the productive initiation complex likely stems from diffusive backtracking of RNAP as shown during elongation [26], although its detection in the presence of Gre proteins appears to be inconsistent with backtracking. Compared to the elongation complex, the reduction of pausing lifetime by the Gre-dependent 3′ RNA cleavage in the initiation complex may be limited because the time that the 3′ RNA end dissociates from the template DNA would be too short to be accessed by Gre proteins when the nascent RNA is ~10 nt or shorter. However, such a short time should be sufficient to partially block the access of small molecule NTPs into the active site, thereby leading to a short-lived pausing.

On the other hand, the short 6–13-nt transcripts that were produced from the promoters with T-rich ITS were significantly reduced by Gre proteins and by the shorter reaction time (Figure 7 and Supplementary Figure S1), clearly indicating that this signal is involved in abortive synthesis by the moribund complex. The T-rich ITS encoding unstable dA-U hybrid allows energetically favored backtracking to form stable dA-dT duplex [47], likely resulting in abortive poly-U release. The backtracked state originating from the latter mechanism should be much more stable than each of the thermally diffusive backtracked states indicated by the low FEINC.

Although further studies are needed to examine whether such in vitro conditions could properly represent the in vivo system, our results suggest that T-rich ITS induces abortive transcription. On the other hand, differently from the T-rich sequence signal, certain repetitive DNA sequences characterized by the low FEINC induce pausing. Both sequence signals presumably induce backtracking, but only in T-rich ITS, the backtracked state become more stable than the non-backtracked state.

### 3.3. Sequence and Nonlocal-Base pair-Stability-Related Contribution to Transcription Productivity

In order to investigate the physicochemical properties of dsDNA that determine the productivity of initiation, we performed a solution NMR experiment using the nine promoters shown in Figure 6B. It is generally accepted that imino protons hydrogen-bonded in dsDNA can exchange with water protons only after opening of the base-pairs [48]. The intensities of imino proton resonances detected by NMR experiment indicate how resistant to opening the base pairs are, and therefore the sum of those intensities through the entire dsDNA can reflect the nonlocal base pair stability, which could be related to dsDNA rigidity.

**Figure 9:**
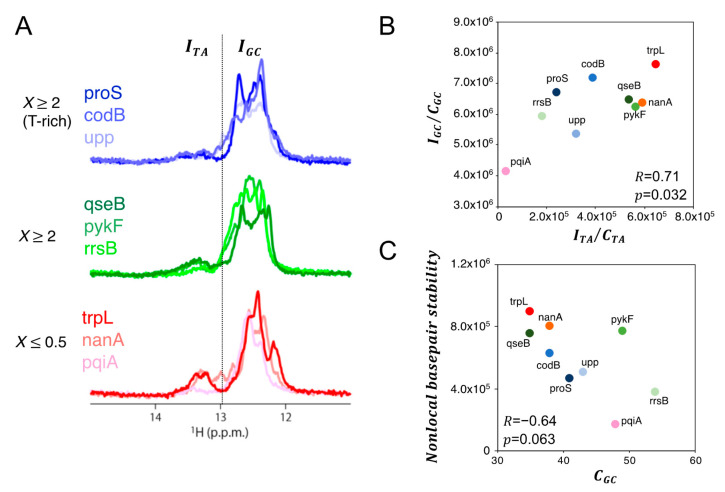
Solution NMR spectroscopy measuring imino proton resonances in 80-bp dsDNA with promoter sequences. (**A**) Proton NMR spectra of the promoter groups shown in Figure 6B. The dotted line represents the boundary between the imino proton signals that are derived from dT-dA base pair (*I_TA_*) and that from dG-dC base pairs (*I_GC_*). Larger integrated signal represents more stable base pairing over dsDNA, which is obtained from slower exchange of the imino proton with water proton. (**B**) Global correlation in the base-content-normalized imino proton resonances between dT-dA base pairs (*I_TA_* /*C_TA_*) and dG-dC base pairs (*I_GC_* /*C_GC_*) among 9 promoter DNA. (**C**) The nonlocal base pair stability tends to negatively correlate with GC contents (*C_GC_*) of promoter DNA. Pearson correlation coefficient *R* and the *p*-value is shown in each graph of panels B and C.

We observed degenerate imino-proton signals derived from individual base pairs in different chemical shift positions (Figure 9A). We assigned those signals to dT-dA base pairs and dG-dC base pairs, respectively, by their dependency on the *GC* content (Supplementary Figure S2). Since the degenerate signal intensities reflect not only the opening dynamics of base pairs but also the content of base pairs in dsDNA, we normalized the signal intensities by dividing them by the base pair content of the dsDNA, focusing only on the dynamics. The normalized signal intensities of dT-dA and dG-dC base pairs globally correlated with each other (Figure 9B), indicating that this value simply reflect nonlocal base pair stability of dsDNA that depends on sequence patterns (such as repetitive sequence elements) rather than the average *GC* content. Thus, we define the value as follows:

$$Nonlocal\;basepair\;stability=\;\frac{I_{TA}}{C_{TA}}+\alpha \frac{I_{GC}}{C_{GC}}$$

where *I_TA_* and *I_GC_* represent the integrated signal intensities of dT-dA and dG-dC base pairs, respectively, and *C_TA_* and *C_GC_* represent *TA* and *GC* contents (%) of the promoter DNA, respectively. A correction coefficient α (~0.033) is obtained by an average value of $\left(\frac{I_{TA}}{C_{TA}}\right)/\left(\frac{I_{GC}}{C_{GC}}\right)$ among nine promoter DNAs tested, which is used for adjusting the different intensity scale between *I_TA_* and *I_GC_*. We noticed that nonlocal base pair stability tends to negatively correlate with GC content (*C_GC_*) through nine promoter DNA (Figure 9C), suggesting that the maintenance of a certain rigidity in promoter DNA might be physiologically significant especially when the *GC* content is low.

**Figure 10:**
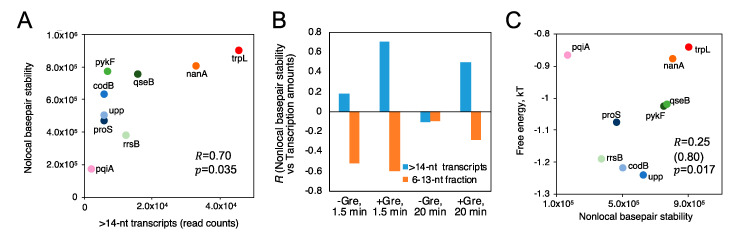
Productivity of transcription depends on nonlocal base pair stability within promoter region. (**A**) Correlation analysis between the nonlocal base pair stability and the number (read counts) of long >14-nt transcripts. The condition of 1.5 min incubation with NTPs in the presence of Gre proteins was used for the analysis. This condition provided the least abortive transcripts from the 9 promoters on average (see Supplementary Figure S1C). (**B**) The higher positive and negative correlations of nonlocal base pair stability with the long and short transcriptions, respectively, are achieved by the lower production of abortive transcripts. The transcription conditions (± Gre proteins, incubation time with NTPs) are indicated at the bottom of each graph. (**C**) Correlation analysis between the dsDNA rigidity and the average FEINC (shown as free energy) calculated for individual promoters within the interval (−40 bp to +40 bp) around TSS. In the panel A and C, Pearson correlation coefficient *R* and the *p*-value is shown. In the panel C, *R* value except pqiA is also shown in parentheses.

We found that nonlocal base pair stability positively correlates with the number (i.e., the read count) of productive >14 nt transcripts (Figure 10A). However, the opposite, negative correlation was observed in the relative fraction of short 6–13 nt transcripts (Figure 10B). The higher the nonlocal base pair stability, the higher the productivity of initiation. This trend can be interpreted as indicating that the introduced nonlocal dsDNA rigidity constitutes one of the key reaction coordinates of the productive initiation. We stress that this parameter is entirely sequence-dependent and it is fundamentally determined by nonlocal physicochemical properties of dsDNA. Consistent with the correlative relationship between FEINC and the number of productive (>14 nt) transcripts (Figure 8A), the nonlocal base pair stability also positively correlates with the FEINC when pqiA was again excluded as an outlier (Figure 10C). The highest correlation for the number of productive transcripts with the nonlocal base pair stability was detected when short (6–13-nt) transcript level was the lowest (by 1.5 min incubation with NTPs in the presence of Gre proteins), while the lowest correlation was obtained when the short transcript level was the highest (by 20 min incubation in the absence of Gre proteins) (Figure 10B). This is similar to the observed correlation between the number of transcripts with FEINC (Figure 8B). These results suggest that nonlocal base pair stability is likely determined to some extent by the presence of repetitive sequence elements.

In summary, our results imply that the pausing-dependent productivity of transcription initiation may be predicted from the two parameters characterizing nonlocal (−40 bp, +40 bp) properties of promoter DNA: (i) an experimentally observable (using NMR) quantity defined here as the nonlocal base pair stability and (ii) a calculated measure characterizing repetitive DNA sequence elements defined here as FEINC. These two parameters are presumably connected to each other in terms of the energetics of transcription initiation.

## 4. Discussion

In this study, we demonstrated that pausing during initiation is induced by nonlocal interactions between RNAP holoenzyme and promoter DNA with the length of ~80 bp. Our results suggest that such nonlocal interactions are modulated by repetitive DNA sequence elements and are quantified by FEINC, which are also connected to nonlocal base pair stability. We identify the stability directly from intensities of imino proton resonances detected by NMR. Therefore, the stability represents an experimentally identifiable reaction coordinate that depends on the physicochemical properties of DNA.

In particular, DNA sequences depleted in repetitive sequence elements (such sequences possess high FEINC, Figure 10C) are characterized by high nonlocal base pair stability. In other words, in the higher stability region, RNAP-DNA binding turns to favor the consensus mode (sequence-specific binding) more than the non-consensus mode (sequence-nonspecific binding). Our key finding here is that the high nonlocal base pair stability (high FEINC) around σ^70^ promoters appears to play a role in preventing initiation from the pathway decreasing the productivity. More precisely, our results suggest that the high nonlocal base pair stability (high FEINC) within the promoter regions can significantly increase the activation energy to the pathways dominated by thermal fluctuations including RNAP sliding on DNA. When base paring is less stable over a wide area including −35 motif, promoter DNA would become more loosely fixed to the σ^70^ subunit of holoenzyme, which allows positional fluctuations (i.e., sliding) between RNAP and DNA. These fluctuations interfere with the σ^70^-specific promoter recognition during the initial binary complex formation, and also induce backtracking in the following stage of the ternary complex. The diffusive backtracking impedes DNA scrunching that has been proposed to facilitate promoter escape [49,50] and forward translocation that is necessary for the next NTP addition to the nascent 3′ RNA end during progressing transcription [51]. This results in pausing. Indeed, in the promoters with a lower nonlocal base pair stability, we observed decreased amounts of promoter-specific transcriptions and increased lifetime of pausing during promoter escape (Figure 10A,B).

We noticed that σ^70^ promoter groups characterized by the low average FEINC (*X* ≥ 2 groups) tend to have more pronounced 6-bp consensus motifs compared to other groups (with intermediate or high average FEINC), especially when RNA is 7 or 8 nt (Figure 4). Thus, in these promoters with the low FEINC, the specific binding of σ^70^ to DNA appears to be enhanced only within the −10 and −35 motifs, maintaining the balance between the consensus and non-consensus modes of protein–DNA binding. This balance may be essential for designing the functional promoters by using only sequence-based properties.

We also identified T-rich ITS as a sequence motif weakly conserved for increasing abortive transcription genome-wide. Such T-rich ITS encodes dA-U hybrid that is less stable than its dsDNA form dA-dT duplex [47], thereby allowing abortive release of oligomeric U by backtracking. As oligomeric U is released, a more stable dA-dT DNA duplex can form. In this sense, the backtracking induced by the signal is energetically favored and is different from that induced by thermal fluctuations on repetitive sequences. We verified that the T-rich ITS causes abortive initiation using our in vitro transcription assay.

### FEINC Predicts Conformational Heterogeneity Of Moribund Complexes

**Figure 11:**
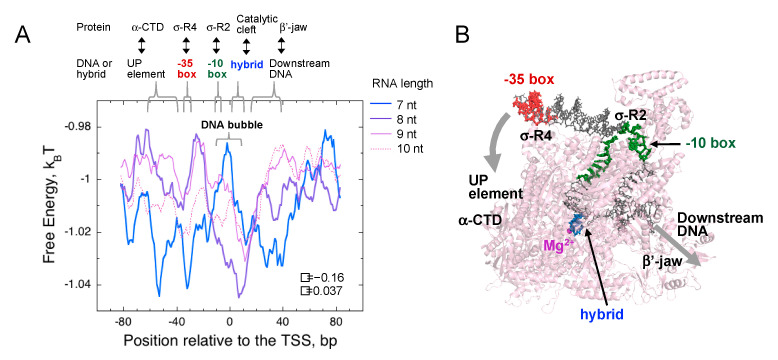
FEINC landscape is altered by the nascent RNA length. (**A**) An opposite trend of the FEINC landscapes is observed between the ternary complexes retaining 7-nt and 8-nt (9 nt and 10 nt) RNA, in the abortive/pausing-enriched group (*X* ≥ 2). Pearson correlation coefficient *R* and the *p*-value in the comparison of the entire free energy indices between the complexes having 7-nt RNA and 8-nt RNA are shown in the graph. (**B**) X-ray crystal structure of the *E. coli* initiation complex with 4-bp RNA-DNA hybrid (PDB ID: 4YLN) [53]. Key interactions between holoenzyme and dsDNA/RNA-DNA hybrid that are described in the panel A are also shown within the structure. DNA and holoenzyme molecules are shown by gray and pink colors, respectively.

One of the key advantages of our statistical approach using the RNET-seq data is that it allows us to analyze the conformational heterogeneity of the ternary complexes in vivo, depending on the length of the nascent RNA retained. Focusing on the abortive/pausing-enriched promoter group (Figure 5, *X* ≥ 2), we found an opposite trend in the landscape of the average FEINC for the complexes retaining 7-nt RNA, as compared to the complexes retaining 8-nt RNA (Figure 11A). In particular, the free energy (i.e., FEINC) of the 7-nt RNA complex was higher compared to the corresponding free energy of the ≥8-nt RNA complex, within the range of the DNA sequence forming the DNA bubble and the RNA-DNA hybrid (~−11 to +7). Conversely, the free energy of the 7-nt RNA complex was lower over the upstream and downstream dsDNA flanking the bubble and the hybrid (Figure 11A,B). Such dsDNA-RNAP interactions include (i) σ region 4 and -35 DNA motif, (ii) the C-terminal domain of the α subunit (α-CTD) and the upstream DNA (UP-element), and (iii) the so-called jaw domain of the β’ subunit and the downstream DNA (Figure 11A,B) [52]. The increased nonconsensus binding mode over the broad range of dsDNA interactions with the protein surface may weaken the strong interaction stemming from σ region 3.2 loop and phosphates of the 5′ nascent transcript as a result of the enhanced sliding between the protein and dsDNA. Indeed, single-molecule studies of different research group have identified a long-lived pause that likely stems from the σ-5′ RNA interaction at the consensus lac promoter when the growing RNA reaches 7-nt [10,11,23]. Interestingly, the pausing observed was involved in 1-bp backtracking [23]. When the RNA was extended to ≥8 nt, the lower free energy region was more localized to the hybrid region and the bubble when compared to that of the 7-nt RNA complex, and was shifted further downstream when the RNA was extended to 9 nt and 10 nt.

Overall, the RNA-length-dependent difference in the FEINC landscapes suggests that the functional significance of the nonconsensus mode in the RNAP-dsDNA-hybrid binding varies depending on the nascent RNA length. In addition to the nonconsensus mode, considerable differences were also observed in the −10/−35 motifs between the complexes of the abortive/pausing-enriched group carrying 7-nt and 8-nt RNAs: 7 nt and 8 nt RNA complexes respectively favor the higher integrity of −35 and −10 motifs (Figure 4). This result suggests that both the nonconsensus and consensus mode of binding cooperatively contribute to determining the RNA-length-dependent conformational heterogeneity.

## 5. Conclusions

Our statistical analysis of transcription initiation using the concept of the non-consensus mode of protein–DNA binding suggests that the fate of the nascent transcript on σ^70^ promoters is determined, at least partially, by repetitive DNA sequence elements around the TSS. Repetitive sequence elements can increase the number of possible conformational states of the ternary complex and cause backtracking. We suggest that certain types of repetitive elements can also decrease the productivity of initiation by lowering nonlocal base pair stability of promoter DNA. Therefore, we argue that the definition of functional promoter sequences should be reconsidered and include quantitative measures to account for the effect of repetitive sequence elements and non-local base pair stability.

Finally, we demonstrate here that concepts of statistical mechanics provide a firm theoretical framework for handling high-throughput sequencing data containing information on microscopic heterogeneity of protein–DNA–RNA ternary complexes. Such microscopic heterogeneity is driven by thermal fluctuations and shaped by the presence of repetitive DNA sequence elements [38]. In this study, we demonstrate that this unique property of macromolecules in aqueous solution can be directly accessed by solution NMR experiments. In the future, combinations of the statistical approach with spectroscopic analyses, which use NMR and emerging terahertz technologies etc. [54], should further help to understand interesting but as yet unexplained phenomena that still remain in the field of transcription.

## Supplementary Materials

The following are available online at https://www.mdpi.com/2218-273X/10/9/1299/s1, **Table S1:** Short RNET-seq reads that were mapped to TSS; **Table S2:** DNA Sequences of the in vitro transcription templates, **Figure S1:** In vitro transcription from 9 different promoters that focus on short transcripts and antisense transcripts, **Figure S2:** Assignment of the imino proton signals.
