# Supplementary material for: Control of Transcription Initiation by Biased Thermal Fluctuations on Repetitive Genomic Sequences
Source: Biomolecules. 2020 Sep 9;10(9):1299. doi: 10.3390/biom10091299 (PMC7564750; doi:10.3390/biom10091299)
Supplement: Supplementary file 1 [file biomolecules-10-01299-s001.pdf]

Supplementary Information

Supplementary text.

In general, single-round transcription assays allows us to identify abortive transcripts as a time-dependent increase in short transcripts up to 20 min. This is because abortive transcripts are very slowly and iteratively synthesized by the binary moribund complex. We compared the short transcript fraction at 20 min incubation with NTPs with that of 1.5 min incubation. The short transcripts increased time-dependently in 7 promoters but decreased in the remaining 2 promoters (nanA and pqiA) (Supplementary Figure S1A), consistent with the abortive/pausing-depleted classification ($X \leq 0.5$) of the 2 promoters. In the trpL promoter of the same $X \leq 0.5$ group, the short transcripts exceptionally increased over time. This inconsistency may be due to the different methods employed in the experiments in vivo and in vitro: we analyzed the nascent transcripts retained in the RNAP in vivo in our previous study [25] but here analyzed total transcripts in vitro because of the weak initiation from some promoters tested in vitro. Such an inconsistency might also be related to substantial anti-sense transcription observed in the experiment in vitro. In fact, the high level of anti-sense transcription was induced by GreAB in pykF promoter (Supplementary Figure S1B), which can explain why the short transcripts were increased by the Gre addition in this promoter while is classified as the abortive/pausing-enriched ($X \geq 2$) group.

Supplementary Table S1. Short RNET-seq reads that were mapped to TSS (see Figure 2A) more than one location (top) and only once (bottom).

Mapped more than one location.

	# of reads	# of mapped reads with perfect match	% of mapped reads	# of mapped reads with perfect match started at TSS	% of mapped reads started at TSS
WT_10nt	774,402	11,301	1.46	3,969	0.51
WT_9nt	515,192	14,341	2.78	2,834	0.55
WT_8nt	310,733	32,489	10.46	4,500	1.45
WT_7nt	236,912	72,590	30.64	9,950	4.20
ΔgreAB_10nt	395,924	8,592	2.17	3,347	0.85
ΔgreAB_9nt	300,441	14,310	4.76	1,712	0.57
ΔgreAB_8nt	222,513	29,609	13.31	3,833	1.72
ΔgreAB_7nt	191,646	60,698	31.67	8,062	4.21

Mapped once.

	# of reads	# of mapped reads with perfect match	% of mapped reads	# of mapped reads with perfect match started at TSS	% of mapped reads started at TSS
WT_10nt	774,402	11,175	1.44	3,959	0.51
WT_9nt	515,192	13,702	2.66	2,806	0.54
WT_8nt	310,733	26,317	8.47	4,361	1.40
WT_7nt	236,912	52,917	22.34	9,498	4.01
ΔgreAB_10nt	395,924	8,358	2.11	3,309	0.84
ΔgreAB_9nt	300,441	13,595	4.53	1,635	0.54
ΔgreAB_8nt	222,513	20,391	9.16	3,679	1.65
ΔgreAB_7nt	191,646	37,778	19.71	7,673	4.00

Supplementary Table S2. DNA Sequences of the in vitro transcription templates.

**Promoter**	**Sequence, 140 bp**
proS	TGAAGTCTTTGCGCTGGAACCGCGTTAAATTCACGCCCTTCTCTTTTGACATTTCTTTTGCACTGGTAAACTAAATCACTT TTTTTTGTCCCAGGCTCGCCTTGAGCCTGTTCTACCTTCCAACTGGAACCGTAACAACA
codB	TTGCACTCATTCATATAAAAAATATATTTCCCCACGAAAACGATTGCTTTTTATCTTCAGATGAATAGAATGCGGCGGATT TTTTGGGTTTCAAACAGCAAAAAGGGGGAATTTCGTGTCGCAAGATAACAACTTTAGCC
upp	ATCTCAAACCGTTATCATTTTGACTAAAGTCAACGAAAAGAATATTGCCGCCTTGAAGAAAGGAGGTATAATCCGTCGAT TTTTTTTGTGGCTGCCCCTCAAAGGAGAAAGAGTATGAAGATCGTGGAAGTCAAACACCC
qseB	TTCATGTTTATTACTCCCTTTAATGTCTGTTTCCGAGCATTTAACAAGATAGTCCTTAACAACTTCTTAAGGGAAAAAAAT AAAATTTAGTGCTGTACAGAGCGCGTTACAACACGGTTTACTGGCAGCAAATACGGTTA
pykF	CCTATCCTTAGAGCGAGGCACCACCACTTTCGTAATACCGGATTCGCTTTCCGGCAGTGCGCCCAGAAAGCAAGTTTC TCCCATCCTTCTCAACTTAAAGACTAAGACTGTCATGAAAAAGACCAAAATTGTTTGCACCA
rrsB	GGGTTCTCCTGAGAACTCCGGCAGAGAAAGCAAAAATAAATGCTTGACTCTGTAGCGGGAAGGCGTATTATGCACACC CCGCGCCGCTGAGAAAAAGCGAAGCGGCACTGCTCTTTAACAATTTATCAGACAATCTGTGT
trpL	CGACATCATAACGGTTCTGGCAAATATTCTGAAATGAGCTGTTGACAATTAATCATCGAACTAGTTAACTAGTACGCAAG TTCACGTAAAAAGGGTATCGACAATGAAAGCAATTTTCGTACTGAAAGGTTGGTGGCGCA
nanA	TGCCACTTTAGTGAAGCAGATCGCATTATAAGCTTTCTGTATGGGGTGTTGCTTAATTGATCTGGTATAACAGGTATAAA GGTATATCGTTTATCAGACAAGCATCACTTCAGAGGTATTTATGGCAACGAATTTACGTG
pqiA	AAAAACCGAAGAAGCCGCCGCGGCAAAAGCAGAAACTGTAAAACGCAGCAGTAGCAAACTAAGCTATAAATTGCAGCG CGAACTGGAGCAGCTACCGCAATTGCTCGAAGATCTGGAGGCGAAGCTGGAAGCCCTACAGA

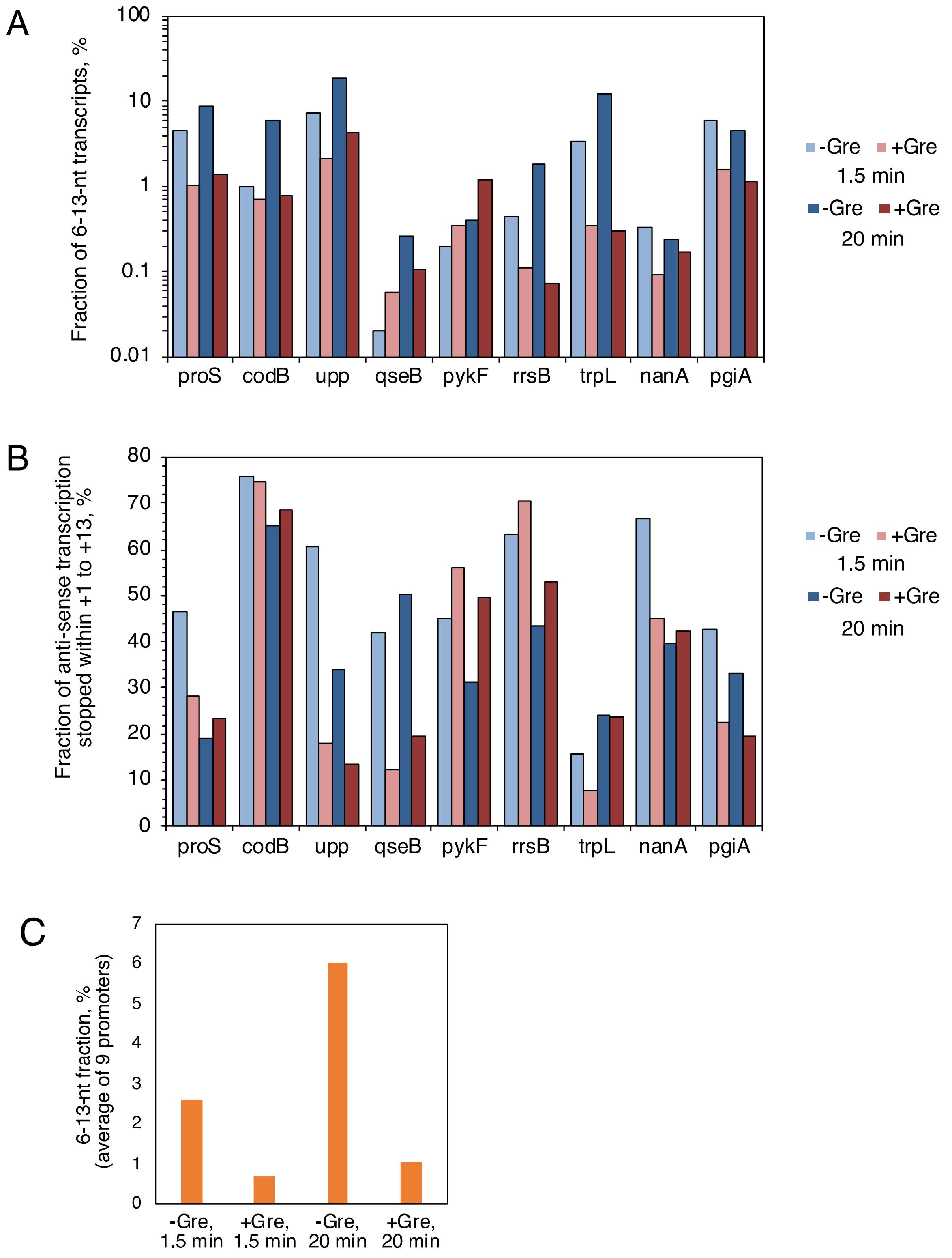

Supplementary Figure S1. In vitro transcription from 9 different promoters that focus on short transcripts and antisense transcripts. (A) Time- and Gre-dependent changes in the fraction of short transcripts. (B) Antisense transcription that is stopped around TSS may affect initiation of sense transcription. See Figure 6B for details about the promoter sequences. (C) The condition of 1.5 min incubation with NTPs in the presence of Gre proteins provided the least abortive transcripts from the 9 promoters on average.

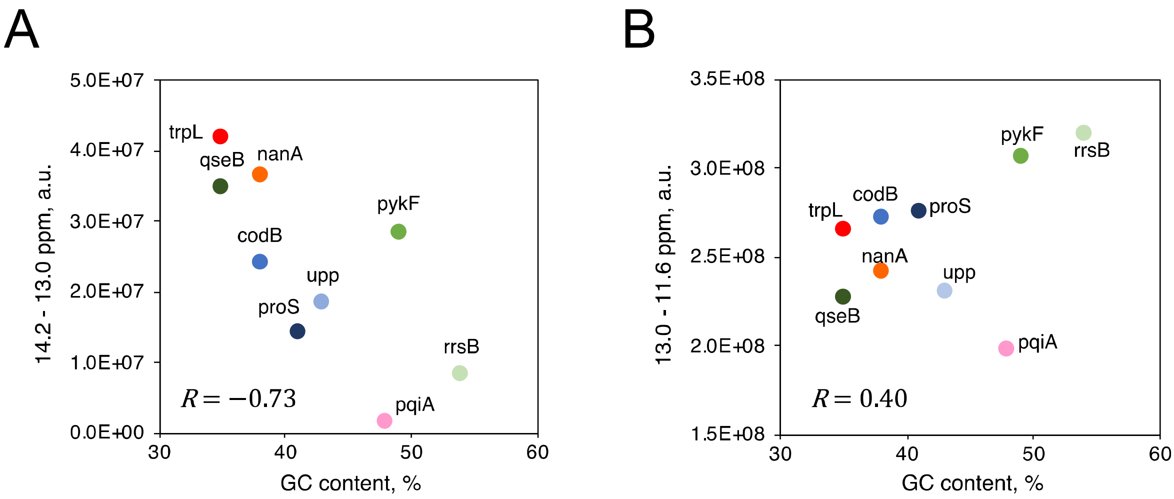

Supplementary Figure S2. Assignment of the imino proton signals. (A) The integrated signals of 14.2-13.0 ppm were assigned to I_{TA} because of the tendency of negative correlation with GC content in the 9 promoter DNAs tested. (B) The remaining integrated signals of 13.0-11.6 ppm were assigned to I_{GC} because of the tendency of positive correlation with the GC content as well as the larger intensities of the signals.
